# Structural Heart Interventions in Women with Congenital Heart Disease

**DOI:** 10.1007/s11886-026-02394-0

**Published:** 2026-08-28

**Authors:** John-Anthony Coppola, Shiva Barforoshi, Jina Chung, Pei-Ni Jone, Aparna Kulkarni, Jennifer Co-Vu

**Affiliations:** 1 Pediatric Heart Center, University of California Davis TICON II suite 210, TICON II suite 210, Sacramento, CA 95817 USA; 2 Harbor UCLA Medical Center, Torrance, CA USA; 3 Lurie Children’s Hospital, Northwestern University Feinberg School of Medicine, Chicago, IL USA; 4 Cohen Children’s Heart Center, Donald and Barbara Zucker School of Medicine at Hofstra/Northwell, New Hyde Park, NY USA; 5 UF Health Congenital Heart Center, University of Florida, Gainesville, FL USA

**Keywords:** Congenital heart disease, Transcatheter intervention, Sex-specific considerations, Women’s health, Health equity

## Abstract

**Purpose of Review:**

To provide a review of current transcatheter interventions in women with congenital heart disease, with a focus on sex-specific considerations in diagnosis, management, and follow-up.

**Recent Findings:**

As the field of pediatric cardiology advances and patients with previously life-limiting diagnoses now survive well into adulthood, there is a growing need to pursue management strategies that improve quality of life. Unfortunately, in the treatment of cardiovascular disease, women have historically been underrepresented in research studies and clinical trials, which may limit the generalizability of available evidence and contribute to disparities in outcomes. Women with congenital heart disease (CHD) are an even more vulnerable population given the relatively low prevalence of CHD. This review discusses several CHD lesions and current trends in diagnosis, management, and follow-up in women. Additionally, it highlights sex-specific considerations in this unique patient population. Future initiatives and applications that may help improve the lifetime care of women with CHD are also reviewed.

**Summary:**

Although there have been increasing efforts to improve the inclusion of women in medical research in recent years, substantial gaps in representation remain. As emerging technologies are increasingly introduced in the CHD field, there is an important opportunity to improve the representation of women in studies evaluating procedural outcomes. Tools such as artificial intelligence and machine learning may help facilitate scalable screening and risk-stratification strategies for women with CHD.

## Introduction

Congenital heart disease (CHD) affects approximately 1 in 100 live births in the United States (US). Advances in prenatal detection, early intervention and procedural and surgical techniques have transformed outcomes, allowing many patients with conditions once considered fatal or terminal, to survive into adulthood and lead productive lives. As a result, there are now more adults living with CHD than children, with the adult CHD population surpassing the pediatric prevalence [1].

While improving both longevity and quality of life has been the central goal of CHD care, this demographic shift necessitates a renewed perspective on CHD management, particularly for female patients. Significant disparities in the care of women with CHD persist [2]. Women with CHD have distinct biological considerations, lifestyle factors, reproductive health concerns, and preventive health screening needs that must be recognized and addressed early in life as part of comprehensive CHD management [3]. Furthermore, women have historically been underrepresented in CHD clinical trials and outcomes research, resulting in important knowledge gaps and limited sex-specific evidence to guide clinical decision-making. In this manuscript, we focus on transcatheter interventions in women with CHD. For clarity, the term *woman* is used to denote biological sex. We will review general considerations, transcatheter approaches to acyanotic and cyanotic heart disease, and future directions aimed at optimizing care and outcomes for women living with CHD.

## Medical Considerations

### Radiation Safety During Cardiac Interventions

Ionizing radiation, even at low doses, is a known carcinogen. Accordingly, any discussion of cardiac catheterization-based interventions must include a clear understanding of the risks associated with radiation exposure (RE). Radiation safety is a particularly important lifelong consideration in women with CHD, who often undergo numerous diagnostic imaging and catheter-based procedures beginning in childhood, with some interventions occurring during pregnancy.

The 2025 American College of Cardiology/American Heart Association (ACC/AHA) guidelines for the management of adults with CHD have a class I recommendation (Level of Evidence B-NR) to limit RE during all procedures to reduce the cumulative lifetime risk of cancer [4]. These guidelines further emphasize the importance of selecting imaging modalities tailored to the specific CHD anatomy and clinical question, favoring non-ionizing modalities when feasible and avoiding computed tomography or fluoroscopy-based interventions in selected circumstances [4].

During a typical pregnancy, background environmental radiation exposure is approximately 0.75–1 mSv (millisievert). From a fetal safety perspective, both the National Council on Radiation Protection and Measurements (NCRP) and the American College of Obstetricians and Gynecologists (ACOG) state that cumulative radiation doses < 50 mGy (milligray) are not associated with miscarriage, congenital malformations, growth restriction, or neurodevelopmental impairment [5]. The NCRP additionally recommends limiting occupational radiation exposure to < 5 mSv during the entire pregnancy. [5] The International Commission on Radiological Protection provides a more conservative threshold, considering exposures < 1 mSv during pregnancy to be safe [6].

Deterministic fetal effects are dose- and timing-dependent. Exposure during the preimplantation period (0–2 weeks after fertilization) at doses of approximately 50–100 mGy may result in embryonic loss, whereas teratogenic effects during organogenesis (weeks 2–8) generally require substantially higher doses (> 100–200 mGy). Neurodevelopmental sensitivity is greatest between weeks 8 and 15, with reported thresholds ranging from 60 to 310 mGy [5, 6] Importantly, radiation doses encountered in contemporary cardiac catheterization and electrophysiology laboratories rarely approach these levels. Lactation and breastfeeding are considered safe following RE, consistent with ACOG guidance [7].

Radiation-minimization strategies are essential for all women undergoing catheter-based interventions and are particularly critical during pregnancy. These include strict adherence to As Low As Reasonably Achievable (ALARA) principles, minimization of fluoroscopy time, use of low frame rates and collimation, avoiding unnecessary cineangiography, and optimization of gantry angles [6] Whenever feasible, alternative imaging and guidance techniques should be prioritized, including transthoracic, transesophageal, or intracardiac echocardiography; ultrasound-guided vascular access; electroanatomic mapping; and zero- or near-zero-fluoroscopy techniques, especially during electrophysiology procedures [4, 8–10].

Adjunctive protective strategies may include tailored shielding technologies that reduce operator scatter radiation (e.g., RADPAD, EggNest), abdominal and pelvic shielding, optimized patient positioning, and coordinated multidisciplinary cardio-obstetric planning [5, 11] Evidence supports that clinically necessary cardiac and structural interventions should not be deferred solely because of radiation concerns during pregnancy, as maternal cardiovascular stability is paramount to fetal well-being. Clear counseling that contextualizes the relative risks of radiation exposure versus procedural delay should be provided in all cases [4, 9, 10].

### Pregnancy Decisions and General Anticoagulation Guidelines in Women with CHD

Contemporary ACC/AHA guidance on anticoagulation in adults with CHD emphasizes individualized shared decision-making and multidisciplinary coordination, with careful consideration of bleeding risk, reproductive plans, and pregnancy status in women [4]. The guidelines caution that commonly used risk-stratification tools, such as the CHA_2_DS_2_-VASc score, may not be applicable to patients with complex CHD, including those with Fontan circulation or Eisenmenger physiology.

Several general principles guide anticoagulation management in this population. Anticoagulation carries a Class I recommendation for women with atrial flutter or fibrillation, prior thromboembolic events, or atriopulmonary Fontan connections. In contrast, anticoagulation is not recommended in Eisenmenger syndrome (Class III) because of the heightened risk of bleeding. Estrogen-containing contraceptives should be avoided in women with increased thrombotic risk, including those with cyanotic CHD or mechanical heart valves.

Guidance regarding anticoagulation in women with mechanical heart valves during pregnancy is based on the 2020 ACC/AHA guidelines for the management of valvular heart disease [12]. These recommendations emphasize shared decision-making that clearly outlines the trade-offs between vitamin K antagonists (VKA), such as warfarin and alternative anticoagulation strategies. Although VKAs are associated with the lowest maternal thrombotic risk, they carry higher risks of miscarriage, fetal loss, and embryopathy—particularly during the first trimester and at doses exceeding 5 mg/day. Three management strategies are commonly considered: continuation of VKA therapy throughout pregnancy, substitution with heparin for the entire pregnancy; or use of heparin during the first trimester followed by VKA therapy during the second and third trimesters.

Detailed recommendations regarding pregnancy surveillance and disease-specific management in women with CHD are available in the most recent ACHD and valvular heart disease guidelines [4, 12].

### Acyanotic Congenital Heart Disease Considerations

#### Sinus Venosus Atrial Septal Defect 

**Fig. 1:**
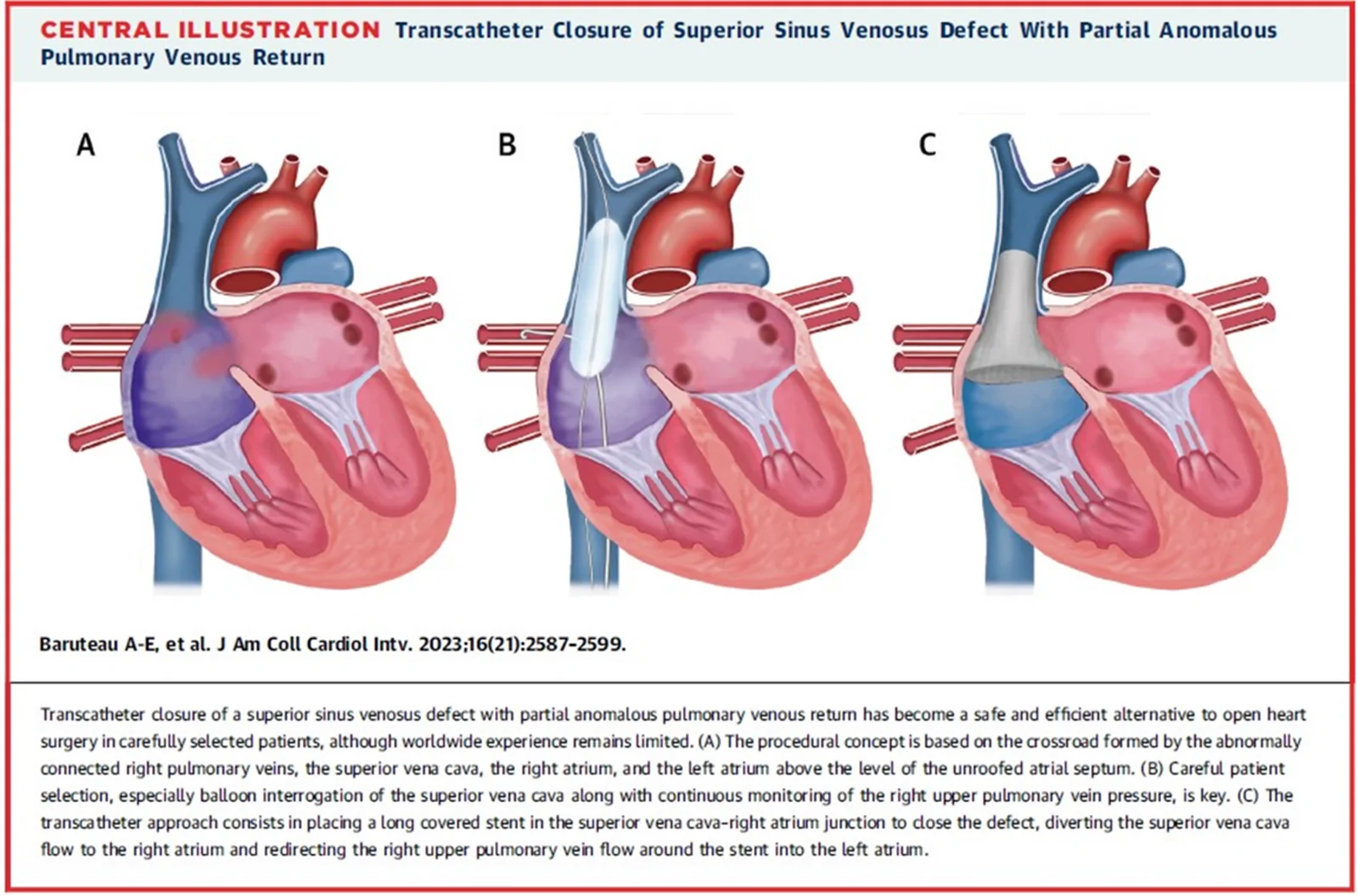
Transcatheter Closure of Sinus Venous Defect. (Reprinted from Baruteau AE et al. JACC Cardiovasc Interv. Nov 13 2023;16(21):2587-2599. doi:10.1016/j.jcin.2023.07.024, with permission from Elsevier) [15]

Sinus venosus atrial septal defects (SVASD) account for approximately 5–10% of all atrial septal defects (ASD) with nearly 90% of these are superior SVASD [13, 14]. There is growing experience with transcatheter closure of superior SVASD with partial anomalous pulmonary venous return (PAPVR). This approach typically employs a covered stent positioned at the superior vena cava (SVC)-right atrial (RA) junction to redirect the SVC flow into the RA while channeling anomalous right-sided pulmonary venous flow into the left atrium (LA) (Fig. 1) [15, 16].

Pre-procedural imaging includes transthoracic echocardiography (TTE), transesophageal echocardiography (TEE), and cardiac computed tomography (CT) to define defect size, PAPVR anatomy, SVC diameter and vertical height. Procedures are performed under general anesthesia with fluoroscopic and TEE guidance. Transseptal puncture permits access the LA and the right upper pulmonary vein (RUPV). A protective balloon may be positioned in the RUPV to preserve pulmonary venous flow during stent deployment.

**Fig. 2:**
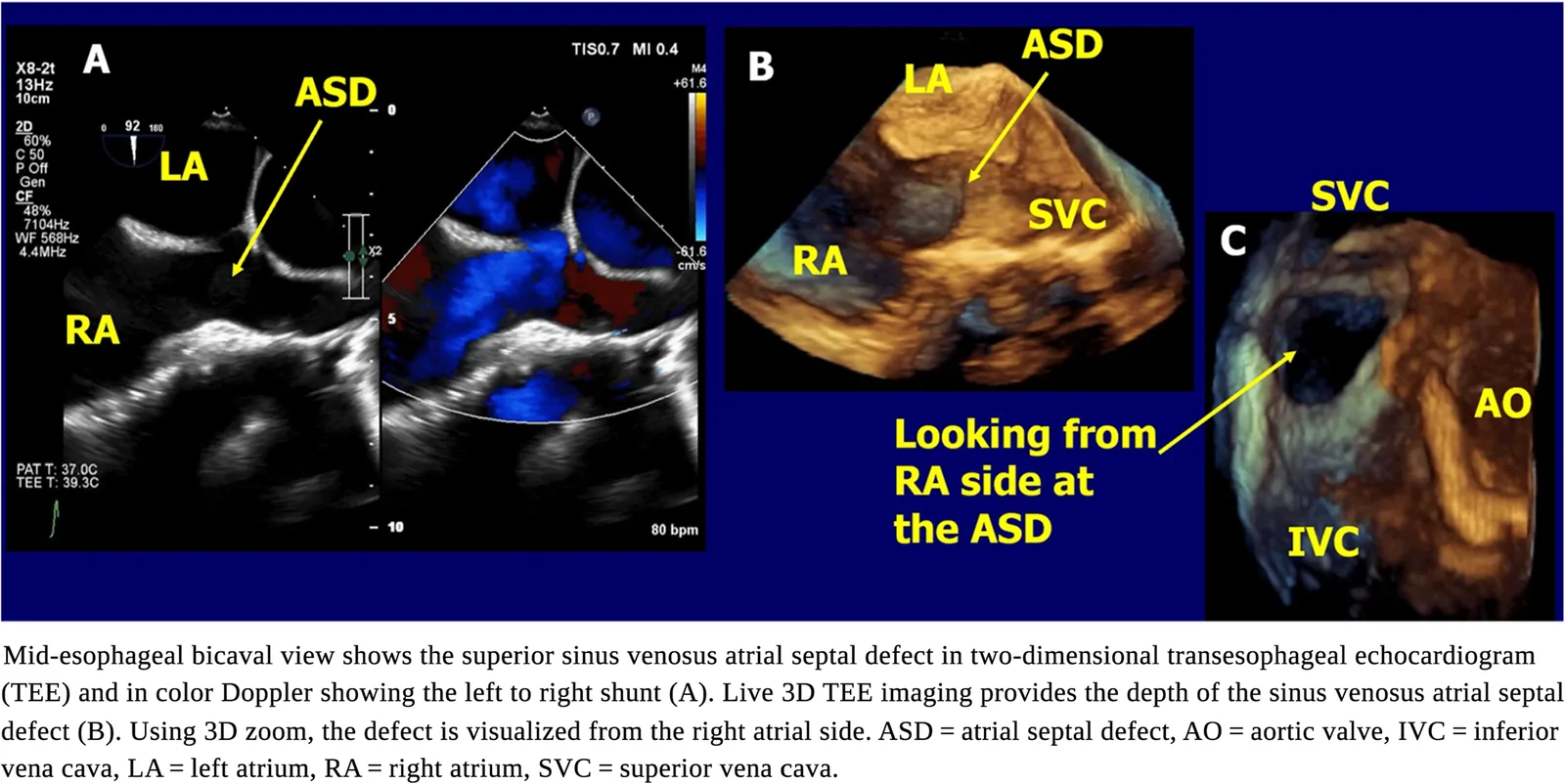
Superior sinus venosus atrial septal defect

Balloon interrogation of the SVC is performed until TEE confirms elimination of the left-to-right shunt without obstruction of the right-sided pulmonary venous return to the LA. The mid-esophageal bicaval view at 90° on 2D TEE and color Doppler demonstrates the SVASD and anomalous pulmonary venous connections (Fig. 2A). Continuous color and pulsed-wave Doppler assessment of right pulmonary venous flow is essential throughout balloon inflation. Live 3D TEE in the 90° bicaval view provides spatial assessment of the depth of the sinus venosus defect (Fig. 2B) and using 3D zoom, the defect can be visualized from the RA side (Fig. 2C). Live 3D TEE provides spatial depth assessment and 3D zoom from the RA and LA perspectives provides critical guidance during covered stent deployment. Post-deployment TEE evaluation includes assessment of SVC patency, residual shunt, and pulmonary venous flow.

Recent data suggest that approximately 80% of superior SVASD cases are anatomically suitable for transcatheter closure [15, 16]. An international registry reported technical success in 97.4% of patients, with major complications occurring in 1.3%, including cardiac tamponade requiring surgical drainage, pacemaker implantation, and pulmonary vein occlusion requiring surgery [17]. Another study reported comparable outcomes with surgical closure, with fewer late complications (12% vs. 23%) and shorter hospital stays following transcatheter closure [18]. Future development of dedicated stent platforms for this specific defect may further improve procedural safety and durability [15]. Overlapping stent strategies may provide additional protection for RUPV drainage [19].

#### Atrial Septal Defect with Pulmonary Hypertension: Fenestrated Occluders

In patients with ASD and pulmonary artery hypertension (PAH), fenestrated occluder devices offer a tailored approach that balances shunt reduction with right ventricular decompression [20]. After at least three months of pulmonary vasodilator therapy, transcatheter closure using a fenestrated device may be considered in patients with Qp/Qs ≥ 1.5 following medical optimization. Pulmonary vasodilators are typically continued post-procedure, along with six months of dual antiplatelet therapy [21].

Available device options include custom-fenestrated Amplatzer devices [21], Occlutech atrial flow regulators (AFR) with predefined fenestration diameters [22], and purpose-designed fenestrated Occlutech ASD devices [23]. Reported outcomes demonstrate reductions in systolic pulmonary artery pressure, improved exercise capacity, increased cardiac index, and resolution of syncope, while maintaining device patency [21, 22]. Carefully selected patients with large ASDs and pulmonary hypertension may therefore benefit from fenestrated transcatheter closure.

### Transcatheter VSD Closure

Ventricular septal defects (VSDs) are the most common type of CHD in pediatrics and the second most common type in adults. Surgical repair via sternotomy and cardiopulmonary bypass has historically been the standard approach. However, transcatheter VSD closure—particularly in Asia—has expanded substantially.

In the United States, the FDA-approved device includes the Amplatzer Muscular VSD Occluder and the Amplatzer Post-Infarct Muscular VSD Occluder is available under an FDA Humanitarian Device Exemption [24, 25]. Other devices used internationally include single- and double-disc systems and coil-based devices such as the Nit-Occlud Lê VSD Coil (PFM, Germany), Table 1. Device selection depends on defect type, location, and associated lesions. Perimembranous and outlet defects pose challenges due to their proximity to the aortic valve and conduction tissue [26].

**Table 1:** Transcatheter Devices Used to Close VSD

Single disk occluders	Double disk occluders
Amplatzer Duct Occluder (ADOI; Abbott, USA)	Amplatzer Duct Occluder II (ADOII; Abbott, USA)
Cocoon Duct Occluder (CDO; Vascular Innovations, Thailand)	Amplatzer Muscular VSD Occluder (AMVO; St Jude Medical, MN, USA)
Cera PDA Occluder (CeDO; Lifetech Scientific, China)	Cocoon VSD Occluders (membranous: CVO-ME; muscular: CVO-MU; Vascular Innovations, Thailand)
Occlutech^®^ Duct Occluder (ODO; Occlutech, Sweden)	Lifetech VSD Occluders (membranous: LVO-MS; muscular: LVO-MU; Lifetech Scientific, China)
	Cera VSD Occluder (Lifetech Scientific, Shenzhen, China)
	MFO (Lifetech Scientific, China)

In adults without CHD, post-MI VSDs have been addressed in the interventional suite, but these are complex and is associated with significant patient and periprocedural risks which are beyond the scope of this paper [27]. The typical approach to closure of VSDs is via an arteriovenous loop, although certain defects may allow for a retrograde approach [28]. Careful imaging and procedural attention to the aortic valve, tricuspid apparatus, and conduction system are essential to minimize complications. Reported adverse events include new or worsened aortic or tricuspid regurgitation and conduction abnormalities, occasionally necessitating surgical intervention [24, 29].

Although most data derive from single-center series, outcomes continue to improve with advances in device technology. Currently, no sex-specific outcome data are available.

#### Aortic Valve Considerations

Congenital aortic valve anomalies—including bicuspid aortic valve (BAV), unicuspid, and quadricuspid morphologies—represent important contributors to aortic valve disease. BAV is the most prevalent congenital cardiac malformation, affecting 0.5–2% of the population with a 3:1 male predominance [30–33]. BAV accounts for 5–20% of transcatheter aortic valve replacement (TAVR) cases in Western cohorts and up to 50% in China [30, 31].

Unlike degenerative tricuspid aortic stenosis— in which women more commonly exhibit fibrotic rather than calcific degeneration—BAV demonstrates predominantly calcific pathology in both sexes [32, 33]. Women with BAV, however, typically have smaller annular and left ventricular outflow tract (LVOT) dimensions. BAV anatomy itself—characterized by annular ellipticity, heavy raphe calcification, asymmetric root geometry, and associated aortopathy—introduces additional procedural challenges [34]. These anatomic features contribute to higher observed rates of paravalvular regurgitation, conduction disturbances requiring permanent pacemaker implantation, prosthesis malposition, stroke, annular rupture, stent underexpansion, and concerns regarding long-term durability following TAVR [30].

Although TAVR is now established across all surgical-risk strata, women remain underrepresented in TAVR cohorts involving BAV (39%) [35]. This disparity likely reflects referral patterns and heightened procedural concerns related to smaller annuli, higher sheath-to-artery ratios, and increased vascular complication risk in women [32, 36]. These considerations highlight the importance of understanding sex-specific anatomic and procedural factors in congenital aortic valve disease [36].

Evidence supporting TAVR in bicuspid aortic stenosis (BAS) remains limited because major randomized TAVR trials (e.g., PARTNER 1–3, CoreValve, SURTAVI, Evolut Low Risk) excluded congenital valve anatomies. Early experience with first- and second-generation valves was associated with high rates of paravalvular leak (13–34%), permanent pacemaker implantation (13–43%), and 1-year mortality (4–18%) [37, 38]. Contemporary results have improved substantially with newer valve technologies and systematic CT-based assessment [39, 40]. In the NOTION-2 trial (*n* = 370; 37% women), the only randomized comparison of TAVR versus SAVR in younger, low-risk patients that included bicuspid aortic valve anatomy (27%; 44% women, predominantly Sievers type 1), overall outcomes were similar between treatment arms. However, BAV patients showed a trend toward higher rates of the primary endpoint, nondisabling stroke, and paravalvular regurgitation compared with SAVR and patient with tricuspid aortic valve.

Sex-specific data for TAVR in patients with BAV remain limited to observational studies and administrative databases. Across cohorts, women demonstrate higher rates of vascular and bleeding complications, largely attributable to smaller iliofemoral artery diameters. In the single-center registry from West China Hospital (*N* = 510, 37% women), women had fewer comorbidities but more vascular (12.9% vs. 4.9%) and bleeding complications (11.1% vs. 5.3%), along with higher residual gradients, although mortality at 30 days and 1 year was similar between sexes [41]. Importantly, bleeding was the only independent predictor of 1-year mortality in women, underscoring the need to minimize intraprocedural bleeding risk.

In an expanded cohort including both bicuspid and tricuspid aortic stenosis (*N* = 1426), BAV morphology did not modify the association between sex and clinical outcomes. Women experienced better overall survival but a higher rate of stroke, whereas men more frequently required pacemaker implantation and valve reintervention [42]. Similarly, in the multicenter Gitto registry (*N* = 980, 37% women), women had lower device success rates and higher rates of periprocedural bleeding but comparable 3-year outcomes. Predictors of adverse events differed by sex, with diabetes, COPD, and severe calcification associated with events in men, and chronic kidney disease being predictive in women [43]. Large administrative cohorts reinforce these trends: Rivera et al. (*N* = 6010, 38.9% women) found similar mortality and major complication rates between sexes, although women exhibited higher unadjusted vascular complication rates [35]. Ullah et al. similarly observed higher unadjusted in-hospital mortality in women (3.2% vs. 2.4%) and increased pacemaker implantation in men (9.8% vs. 6.3%), the latter likely related to the use of larger prostheses use and prolonged conduction system compression [44].

As TAVR expands to younger patients with longer life expectancies, anatomic feasibility and exclusion criteria are critical to procedural planning and outcomes. Current optimization strategies include CT-based sizing and anatomic modeling, tailored procedural approaches such as pre- and post-dilation and higher valve implantation, and proactive coronary access planning—particularly in women, who more often have smaller annular dimensions, lower coronary heights, and smaller sinus dimensions. In younger patients with BAV anatomy, anticipation of future valve-in-valve procedures further underscores the importance of adjunctive leaflet-modification techniques, including BASILICA and UNICORN, to enhance procedural safety [45, 46].

Table 2 highlights a summary of the current research. Further prospective studies and randomized data are needed to better define sex-specific outcomes in congenital valve disease.

**Table 2:** Summary of key trials and registries evaluating sex-specific characteristics and outcomes of TAVR in patients with congenital aortic valve disease

Study Type	Trial/Registry (Year)	Design/Comparator	*N*	% Women	Population/Valve Type	Sex-specific outcomes reported	Key sex-specific findings
**Early observational**	** Mylotte 2014**	Multicenter registry	139	33%	BAV AS	No	
**Early observational**	** Jilaihawi 2015**	Multicenter observational	139	32%	BAV AS	No	
**Early observational**	** Perlman 2016**	Multicenter registry	79	30%	BAV AS	No	
**Randomized**	**NOTION-2** **(Jørgensen** **2024)**	TAVR vs. SAVR	370	37%	Low-risk AS (27% BAV)	No	
**Single-center registry**	** He 2022 (West China Hospital)**	Observational registry	510	37%	BAV AS	Yes	Women had higher vascular and bleeding complications; mortality at 30 days and 1 year was similar
**Administrative database**	** Ullah 2023**	National inpatient cohort	7,000+	40%	BAV + TAV AS	Yes	Women had higher in-hospital mortality; men had higher rates of permanent pacemaker implantation
**Multicenter registry**	**Gitto Registry 2024**	Multicenter observational	980	37%	BAV AS	Yes	Women had lower device success and higher periprocedural bleeding; 3-year outcomes were similar
**Administrative database**	** Rivera 2024**	National administrative cohort	6,010	38.90%	BAV TAVR	Yes	Mortality and major complication rates were similar by sex; women had higher unadjusted vascular complication rates
**Multicenter cohort**	** He 2025**	Observational cohort	1,426	38%	BAV + TAV AS	Yes	Women experienced better survival but higher stroke rates; men more frequently required pacemaker implantation and valve reintervention

*BAV* Bicuspid aortic valve, *AS* aortic stenosis, *TAV* transcatheter aortic valve, *TAVR* transcatheter aortic valve replacement

#### Coarctation of the Aorta (CoA)

Sex differences influence the presentation and long-term management of CoA. However, despite increasing representation of women, sex-specific outcome analyses in CoA remain limited, as most studies were not designed or powered to evaluate sex-stratified endpoints. Modern registries - such as Mayo Clinic databased analyzed by Egbe et al. *N* = 214, 43% women and the cohort reported by Beauchesne et al. *N* = 50, 100% women - demonstrate that women with CoA more frequently present with associated left-sided obstructive lesions such as BAV40 and exhibit a higher prevalence of chronic hypertension and pregnancy-related cardiovascular complications, often reflecting later diagnosis and diagnostic delay [47, 48]. Recent interventional data, including multi-institutional studies of covered and bare-metal stents, show no major sex differences in procedural success or early hemodynamic improvement, although women continue to experience higher rates of vascular access complications due to smaller iliofemoral diameters and higher sheath-to-artery ratios [49, 50].

Modern stent-era outcomes from the COAST (*N* = 105, 30.5% women), and COAST II (*N*= 158, 35% women) extension studies demonstrate sustained gradient relief with low aneurysm rates, and no significant sex-related differences in reintervention or stent durability, although women remained underrepresented (~ 35–40%) [49, 51].). Emerging technologies such as growth-adaptable stents (e.g. Minima expandable stent) designed to be implanted in childhood and subsequently dilated to adult diameters, represent a major advancement in lifetime CoA management by reducing the need for reintervention and enabling growth-accommodating therapy from pediatric through adult stages. Early observational data suggest that these growth-adaptable stents may further minimize long-term complication risks and could help narrow sex-related disparities in vascular access and reintervention [52].

Newer ACHD registry data (IMPACT, CONCOR) likewise demonstrate similar medium-term survival and blood pressure control across sexes following transcatheter or surgical CoA repair, while emphasizing persistent sex-specific vulnerabilities, particularly hypertension and aortopathy in women, that warrant tailored surveillance strategies [53, 54]. Overall, these contemporary data suggest that while procedural outcomes are largely comparable, sex-specific patterns in comorbidities and vascular anatomy continue to influence long-term risk and management in repaired CoA.

### Cyanotic Congenital Heart Disease Considerations

#### Single Ventricle Physiology and Fontan Circulation

Patients with single ventricle physiology undergo staged palliation culminating in the Fontan procedure. Although the 10-year transplant-free survival approaches 90% and 30-year survival approaches approximately 80%, significant long-term morbidity persists [55].

Fontan physiology results in elevated central venous pressure and reduced cardiac output [55]. The complications associated with Fontan include protein losing enteropathy (PLE), plastic bronchitis, baffle stenosis, or low cardiac output symptoms. Lymphatic dysfunction contributes significantly to PLE and plastic bronchitis, and interventional lymphatic embolization has emerged as a promising therapeutic strategy [56]. The ability to manage these complex conditions via an interventional approach can have contribute to better outcomes and improved quality of life for patients with complex single ventricle anatomy.

Older patients may develop atrioventricular valve dysfunction, particularly those with unbalanced AVSD or systemic right ventricles. [57] Transcatheter valve-in-valve implantation via either hybrid or fully percutaneous approaches has been reported [58–61].

Beyond the Fontan population, adult patients with complex CHD who have atrioventricular valve dysfunction may benefit from newer advanced catheter technologies such as MitraClip and TriClip. As patients age, multiple factors that may increase surgical complexity, including multiple redo operations, bleeding risk, ventricular dysfunction, and other comorbidities. ACHD interventionalists have increasingly adapted these technologies for use in patients with complex CHD anatomies [62–64].

#### Tetralogy of Fallot – Transcatheter Pulmonary Valve Replacement

Transcatheter pulmonary valve replacement (PVR) is performed in 70–71% of patients with repaired Tetralogy of Fallot (TOF) patients [65, 66]. There are several transcatheter pulmonary valve options with favorable short- to mid-term outcomes [65, 66]. The current FDA-approved and available devices are listed in Table 3 [67]. The technical success rate is high across all valve types across international registries [68, 69]. Transcatheter PVR is associated with shorter hospital stays compared with surgical valve replacement and may reduce mortality risk by 36% compared to surgical replacement [66, 70]. However, transcatheter PVR carries a threefold higher risk of infective endocarditis compared with surgical replacement [70]. Right ventricular function does not consistently improve on mid-term follow-up [71], and tricuspid valve injury occurs in ~ 3% of cases [68]. No sex-specific data regarding outcomes, reintervention rates, or complications currently exist, although valves used in women are usually smaller compared to those used in men [67, 68].

**Table 3:** The current available transcatheter pulmonary valves

Balloon Expandable Valves	Self-expanding Valves (for larger native/patched RVOTs)
Melody Valve (Medtronic) – maximum diameter 22 mm, designed for conduits and bioprosthetic valves	Harmony valve (Medtronic) – FDA approved in 2022
Sapien Valves (Edwards Lifesciences) – Xt and S3 models up to 29 mm diameter, FDA- approved for dysfunctional conduits and surgical bioprosthetic valves	Alterra Adaptive Prestent (Edwards Lifesciences) – FDA approved, configured as landing platform for Sapien S3
	Venus P-valve, Pulsta valve, and Med-Zenith PT valve are used outside the United States

#### Women in Research and Future Directions

Women historically have been underrepresented in cardiovascular research and the reasons are postulated to be multifactorial [72]. A recent cross-sectional study reviewing research over a 20-year span demonstrated underrepresentation of women in cardiology, pediatrics and infectious diseases, particularly in clinical trials [73]. Additionally, despite ongoing advances in the non-congenital cardiovascular field, studies involving patients with CHD remain limited. Strategies to improve representation in CHD studies include leveraging long-term patient-provider relationships, utilizing specialized research nursing teams, and offering virtual recruitment strategies may aid in improving the number of female participants [74], all of which may help increase female participation.

Artificial intelligence (AI) and machine learning models may enhance screening and risk stratification in women. Chung et al. reviewed recent areas in cardiovascular care in which AI and predictive models were utilized to assess various disease states [75]. Additionally, there are reports of utilizing machine learning to predict sex discordance scores, to potentially improve screening in women with cardiovascular disease [76]. A recent study in Nigeria showed that AI-guided screening using a digital stethoscope and ECG improved detection pf cardiomyopathy in women [77]. However, AI models depend on representative datasets, underscoring the need for improved female recruitment.

Bioabsorbable materials represent another emerging frontier. Currently, all transcatheter devices are non-biodegradable. Early animal and human feasibility studies using bioabsorbable materials demonstrate promising safety and efficacy for transcatheter defect closure. Matsuzki and colleagues recently published a feasibility study in a sheep model, investigating an ASD device utilizing Poly(L-lactide-co-epsilon-caprolactone) [78]. Although such technology remains far from routine use in humans, this represents a very important step in the management of patients with CHD. In addition to ASD devices, there have been two recent studies evaluating bioabsorbable VSD occluders. Wang et al. presented the use of poly-L-lactic acid (PLLA) fabric in a multicenter randomized controlled trial, demonstrating noninferiority to traditional nitinol devices [79]. Huang et al. reported the use in pediatric patients of a fully bioabsorbable perimembranous device made of poly-p‐dioxanone (PDO) threads and internal poly(l‐lactic acid) (PLLA) biodegradable flow‐reducing membrane [80], with several other studies reporting complete device resorption by 3 years [81]. As transcatheter management expands, bioabsorbable materials may improve long-term quality of life by eliminating permanent non-biodegradable intracardiac implants.

## Conclusion

As the population of adults with CHD continues to expand, durable and less invasive therapeutic strategies are essential to preserve quality of life. Transcatheter interventions provide effective alternatives to repeat surgical procedures. It is imperative that women with CHD are adequately represented in device development, clinical trials, and longitudinal follow-up studies. Optimizing outcomes for these patients requires sex-specific surveillance strategies, durable management approaches, and thoughtful consideration of reproductive and lifestyle factors. Ensuring equitable representation will be essential for advancing the care of all patients with congenital heart disease.

## Key References

- Rosenthal E, Qureshi SA, Sivakumar K, et al. Covered Stent Correction for Sinus Venosus Atrial Septal Defects, an Emerging Alternative to Surgical Repair: Results of an International Registry. Circulation. Mar 18 2025;151(11):744–756
  - ○ Findings from this study show increase in the closure of Sinus Venous ASDs in the cardiac catheterization suite as an option for adult patients with congenital heart disease.
- Kunadian V, Pompei G, Dasgupta I, et al. Strategies to enhance recruitment of female participants to cardiovascular research: a joint British Cardiovascular Societies' consensus document in collaboration with the British Heart Foundation Clinical Research Collaborative. Heart. Aug 12 2025;111(17):e3. doi:10.1136/heartjnl-2024–325545
  - ○ Findings from this manuscript discuss the significant inequity of women’s representation in medicine and strategies to mitigate this disparity.

## Data Availability

No datasets were generated or analysed during the current study.
